# Fatigue-Associated Alterations of Cognitive Function and Electroencephalographic Power Densities

**DOI:** 10.1371/journal.pone.0034774

**Published:** 2012-04-13

**Authors:** Masaaki Tanaka, Yoshihito Shigihara, Masami Funakura, Etsuko Kanai, Yasuyoshi Watanabe

**Affiliations:** 1 Department of Physiology, Osaka City University Graduate School of Medicine, Abeno-ku, Osaka City, Osaka, Japan; 2 Digital & Network Technology Development Center, Panasonic Corporation, Kadoma City, Osaka, Japan; 3 Center for Molecular Imaging Science, RIKEN, Chuo-ku, Kobe City, Hyogo, Japan; University of British Columbia, Canada

## Abstract

Fatigue is a common problem in modern society. We attempted to identify moderate- to long-term fatigue-related alterations in the central nervous system using cognitive tasks and electroencephalography (EEG) measures. The study group consisted of 17 healthy male participants. After saliva samples were collected to measure copy number of human herpesvirus (HHV)-6 DNA to assess the level of moderate- to long-term fatigue, subjects were evaluated using EEG, with their eyes open for 2 min, then closed for 1 min sitting quietly. Thereafter, they completed cognitive task trials to evaluate simple selective attention for 3 min (Task 1) and conflict-controlling selective attention for 6 min (Task 2, which included Stroop trials). The percent error of Task 2 for Stroop trials was positively associated with the copy number of saliva HHV-6 DNA, although the simple selective attention measures in Task 1 did not differ significantly. EEG power densities (especially the alpha power density) during the eye-closed condition were negatively associated with the saliva HHV-6 DNA level. Impaired high-level information processing such as that required for conflict-controlling selective attention in the central nervous system may be a characteristic feature of moderate- to long-term fatigue.

## Introduction

The sensation of fatigue refers to the feeling that people may experience after or during prolonged periods of activity [1]. It is a common problem in modern society. In Japan, more than half of the general adult population complains of fatigue [2]. Acute fatigue is a normal phenomenon that disappears after a period of rest. In contrast, chronic fatigue is sometimes irreversible and the compensation mechanisms that are useful in reducing acute fatigue are not effective for chronic fatigue. Since moderate- to long-term fatigue decreases efficiency in the performance of normal daily activities, it is of great interest to clarify the mechanisms underlying this kind of fatigue and to develop quantitative methods to evaluate levels of moderate- to long-term fatigue and effective therapies to treat it.

Although there have been several objective biomarkers for acute fatigue [3], there have been no objective biomarkers for moderate- to long-term fatigue until recently [4]. The human herpesvirus (HHV)-6 established life-long latency in the macrophage and kept a fairly stable intermediate stage between latency and reactivation, and is shed in saliva. The viral reactivation is indicated by a high copy number of HHV-6 DNA measured by using real-time PCR method. The viral reactivation was triggered by work-induced moderate- to long-term fatigue and the copy number of saliva HHV-6 DNA was decreased after a resting period of approximately one week, suggesting that a high copy number of HHV-6 DNA is associated with moderate- to long-term fatigue. The biomarker, saliva HHV-6 DNA, made it possible to assess the level of moderate- to long-term fatigue.

Since a relationship between chronic fatigue and changes in the central nervous system has been emphasized [5], it would be of great importance to identify neural alterations associated with mild- to long-term fatigue. However, although acute fatigue-related changes in cognitive performance (for example, [6]) and electroencephalography (EEG) variables (for example, [7]) have been widely reported, moderate- to long-term fatigue-related changes in these measures have not been identified. The aim of our study was therefore to identify moderate- to long-term fatigue-associated alterations of cognitive functions and EEG variables using the novel biomarker, saliva HHV-6 DNA.

## Methods

### Participants

Seventeen healthy male volunteers [29.4±10.7 years of age (mean ± SD); age range, 21 to 55 years of age] were enrolled in this study (Table 1). Participants having a history of medical illness, taking chronic medications or supplemental vitamins, current smokers, or those with a body weight less than 40 kg were excluded based on our previous studies [3], [8]–[11]. The general health of each participant was also assessed by physical examination by a medical doctor. The study protocol was approved by the Ethics Committee of Osaka City University, and all participants gave written informed consent for participation in the study.

**Table 1:** Characteristics of study participants.

Age (years)	29.4±10.7
HHV-6 DNA (copies/ml)	3.22±0.97
Task performance	
Percent error of Task 1	2.06 (0.63–3.59)
Percent error of Task 2	3.88 (1.73–7.10)
Percent error of Task 2 for Stroop trials	2.47 (1.70–6.40)
Percent error of Task 2 for non-Stroop trials	3.53 (2.09–7.98)
EEG variables	
O1	
Eyes open	
beta (µV^2^)	0.98±0.39
alpha (µV^2^)	1.11±0.51
theta (µV^2^)	1.24±0.35
delta (µV^2^)	1.85±0.71
Total (µV^2^)	5.18±1.39
Eyes closed	
beta (µV^2^)	1.51±0.51
alpha (µV^2^)	3.12±0.98
theta (µV^2^)	2.11±0.72
delta (µV^2^)	1.92±0.66
Total (µV^2^)	8.66±2.48
Alpha attenuation coefficient	3.51±2.46
O2	
Eyes open	
beta (µV^2^)	1.12±0.41
alpha (µV^2^)	1.22±0.51
theta (µV^2^)	1.42±0.38
delta (µV^2^)	2.00±0.85
Total (µV^2^)	5.76±1.57
Eyes closed	
beta (µV^2^)	1.56±0.53
alpha (µV^2^)	3.09±0.92
theta (µV^2^)	2.17±0.66
delta (µV^2^)	2.13±0.77
Total (µV^2^)	8.96±2.40
Alpha attenuation coefficient	2.86±1.17

HHV-6, human herpesvirus-6; EEG, electroencephalographic.

Data are shown as mean ± SD or median (inter-quartile range).

### Experimental design (Figure 1)

**Figure 1:**
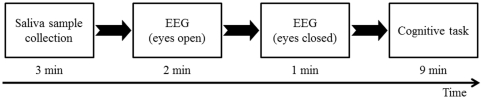
After visit, saliva samples were firstly collected for 3 min. Then, participants were evaluated using electroencephalography (EEG), with their eyes open for 2 min and then closed for 1 min sitting quietly. Finally, to evaluate cognitive functions, they performed cognitive task trials for 9 min.

For 1 day before their single experimental session, participants refrained from intense mental and physical activities, consumed a normal diet and beverages (excluding caffeinated beverages), and maintained normal sleeping hours. The day before their experimental session, participants finished dinner by 21:00, fasted overnight, and then had breakfast before arriving for the experiment. Upon each participant's arrival at the laboratory, saliva samples were collected for 3 min, because the saliva HHV-6 DNA level might be affected by performing EEG or cognitive task trials. After saliva collection, participants were evaluated using EEG, with their eyes open for 2 min and then closed for 1 min, both while sitting quietly. Finally, to evaluate cognitive functions, participants performed cognitive task trials for 9 min. The study was conducted in a quiet temperature- and humidity-controlled environment.

### Cognitive tasks

The cognitive task presentation consisted of traffic lights (a Japanese letter, which means blue or red, was placed on either blue or red lights), traffic signs for walkers (right or left), and turns (right or left) shown on a personal computer screen. The participants performed Task 1 for 3 min and Task 2 for 6 min. In Task 1, subjects were told to press the right button with their right middle finger if the blue traffic light was presented regardless of traffic signs for walkers or turns; if the red traffic light was presented, they were told to press the left button with their right index finger. In Task 2, subjects had to judge whether the target letter presented at the center of a traffic light was blue or red. If the letter meant blue in Japanese, regardless of the color of the traffic light or traffic signs for walkers or turns, they were to press the right button with their right middle finger, otherwise, they were to press the left button with their right index finger. In these tasks, each trial was presented 100 ms after pressing either of the buttons. During the task period, blue or red trials, traffic signs for walkers (right or left), and turns (right or left) were presented randomly, and the occurrence of each color and type of sign was equal. In Task 2, the Stroop trials (mismatching the color of the traffic light with the letter) and the non-Stroop trials (matching the color of the traffic light with the letter) were presented with equal frequency. Subjects were instructed to perform the task trials as quickly and as correctly as possible. The results of each cognitive task trial, that is, a correct response or error, was continuously presented on the computer monitor.

**Figure 2:**
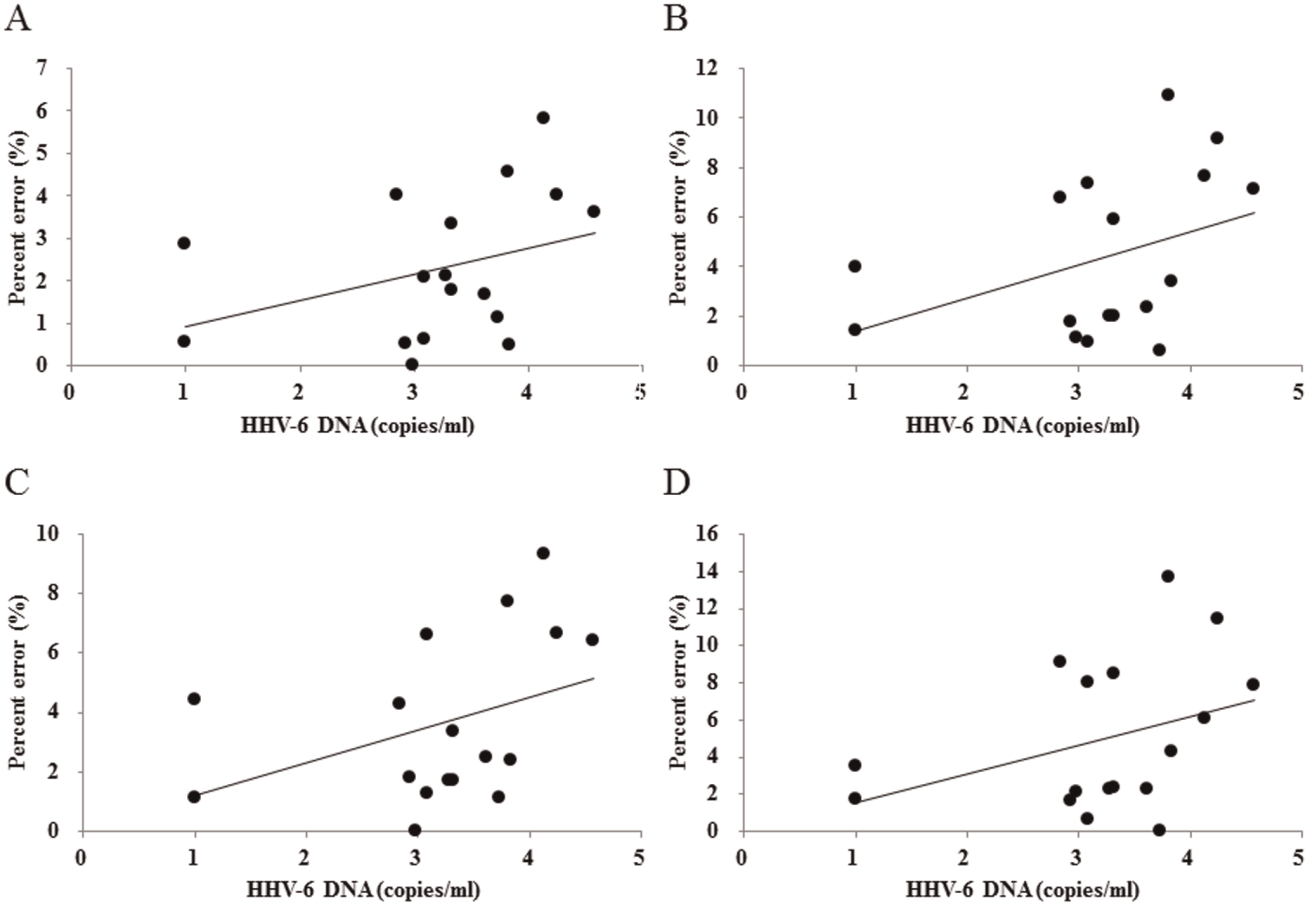
Relationships between moderate- to long-term fatigue and task performances. Correlations between copy number of saliva human herpesvirus-6 (HHV-6) DNA and percent errors of Task 1 (A), Task 2 (B), Task 2 for Stroop trials (C), and Task 2 for non-Stroop trials (D) are shown (n = 17). Linear regression lines are displayed.

### Electroencephalography

EEG measures were made using an EEG system (Neurofax μ EEG-9100; Nihon Kohden Corporation, Tokyo, Japan). Four electrodes (Ag/AgCl) were attached to the head skin, from positions Fp1, Fp2, O1, and O2. All electrodes were referenced to linked earlobes. Electrode impedance was maintained at levels below 5 kΩ during the experiment. The EEG was amplified with a 0.3-s time constant and a 120-Hz low-pass filter, and sampled at 500 Hz. Prior to frequency analysis, all EEG data were divided into epochs with a duration of 1 s. We deleted any epochs with an amplitude higher than 50 µV at Fp1 or Fp2, in order to eliminate eye-blink effects. After artifact detection, the data were subjected to a fast Fourier transform, and spectral power was determined in four frequency bands, delta (1–4 Hz), theta (4–8 Hz), alpha (8–13 Hz), and beta (13–25 Hz), for each participant, electrode, and epoch. The power densities in each frequency band for each participant, averaged across epochs and across frequencies within a band, were log-transformed (ln) for normalization [12].

### Saliva sample analyses

Saliva samples for the analysis of HHV-6 DNA were collected in a tube (Salivette; Sarstedt, Rommelsdorf, Germany) and kept on ice until centrifuged at 1700 g for 5 min at 4°C. These supernatants were stored at −80°C until analyzed. Assays of HHV-6 DNA were performed by Japan Tobacco Inc. (JT; Tokyo, Japan). Values of saliva HHV-6 DNA (copies/ml) were log-transformed (log10).

### Statistical analyses

Correlation analyses were performed to identify fatigue-associated alterations in cognitive performance and EEG variables. Non-parametric Spearman's correlation analyses were used to assess the relationships between copy number of saliva HHV-6 DNA and cognitive performance. Pearson's parametric correlation analyses were used to evaluate the relationships between saliva HHV-6 DNA levels and EEG variables. All p values were two-tailed, and values less than 0.05 were considered to be statistically significant. Statistical analyses were performed using SPSS 17.0 software package (SPSS, Chicago, IL).

**Table 2:** Relationship between copy number of saliva human herpesvirus-6 DNA and electroencephalographic variables.

	R	*p* value
O1		
Eyes open		
beta	−0.149	0.568
alpha	−0.382	0.131
theta	−0.289	0.261
delta	−0.132	0.615
Total	−0.319	0.212
Eyes closed		
beta	−0.347	0.172
alpha	−0.562	0.019
theta	−0.517	0.034
delta	−0.295	0.250
Total	−0.522	0.031
Alpha attenuation coefficient	0.128	0.624
O2		
Eyes open		
beta	−0.241	0.351
alpha	−0.301	0.240
theta	−0.114	0.663
delta	0.038	0.886
Total	−0.168	0.519
Eyes closed		
beta	−0.394	0.118
alpha	−0.572	0.016
theta	−0.397	0.115
delta	−0.084	0.748
Total	−0.441	0.076
Alpha attenuation coefficient	−0.010	0.970

R, Pearson correlation coefficient.

## Results

Correlations between copy number of saliva HHV-6 DNA and task performances are shown in Figure 2. Although the percent errors of Task 1 (Figure 2A) and Task 2 for non-Stroop trials (Figure 2D) did not reach statistical significance (Spearman's correlation coefficient = 0.386; p = 0.126; Spearman's correlation coefficient = 0.391; p = 0.121, respectively), Task 2 showed a trend toward a positive association (Spearman's correlation coefficient = 0.478; p = 0.052) (Figure 2B). Task 2 for Stroop trials was positively associated with the copy number of saliva HHV-6 DNA (Spearman's correlation coefficient = 0.482; p = 0.04997) (Figure 2C).

Relationships between the copy number of saliva HHV-6 DNA and EEG variables are summarized in Table 2. At O1 or O2, beta, alpha, theta, delta, or total (beta plus alpha plus theta plus delta) EEG power densities during the eye-open condition were not associated with the saliva HHV-6 DNA level. In addition, at O1 or O2, alpha attenuation coefficients, calculated as the ratio of alpha power density during the eye-closed condition to that during the eye-open condition [14], were not associated with the saliva HHV-6 DNA level. At O1, total, alpha, and theta power densities during the eye-closed condition were negatively associated with the saliva HHV-6 DNA level. Moreover, At O2, the alpha power density during the eye-closed condition was negatively associated with the HHV-6 DNA level and the total power density showed a trend toward a negative association with the saliva HHV-6 DNA level.

Relationships between age and the EEG variables were not identified. In addition, there were no effects of age on the results of this study (data not shown).

## Discussion

We found that the percent error of Task 2 for Stroop trials was positively associated with the copy number of saliva HHV-6 DNA. In addition, EEG power densities (especially the alpha power density) during the eye-closed condition were negatively associated with the saliva HHV-6 DNA level, though not during the eye-opened condition. Relationship between age and the copy number of saliva HHV-6 DNA was not identified (R = −0.163; p = 0.531). Upon opening one's eyes, eye blinks and eye movement artifacts appear. Thus, we can expect to see weaker relations between moderate- to long-term fatigue and EEG variables during the eye-opened condition than the eye-closed condition. Impaired conflict control and decreased EEG alpha power density during the eye-closed condition were characteristic features of moderate- to long-term fatigue.

Recently, we found that acute mental fatigue caused impaired task performance on Stroop trials. In addition, the fatigued condition induced by sleep deprivation caused reduced performance on the Stroop Color-Word test [14], [15]. In patients with chronic fatigue syndrome, a human model of severe fatigue [16], performance on the Stroop Color-Word test was impaired [17]–[20], and the error was associated with self-reported fatigue levels in patients with depression [21]. Therefore, impaired conflict-controlling selective attention may be a common feature of fatigue-related conditions and diseases.

During the Stroop trials, color and word dimensions activate the associated responses, resulting in conflict between the activated responses and an increased likelihood of error [22]. This conflict is proposed to activate a conflict monitor in the anterior cingulate cortex (ACC), which in turn engages the control function in the dorsolateral prefrontal cortex (DLPFC). This engagement of the DLPFC increases attention on subsequent trials, resulting in improved performance [23]. Because impairments in the ACC and DLPFC have been reported to be associated with fatigue [24]–[33], moderate- to long-term fatigue might cause a deterioration in conflict-controlling selective attention through impaired functions in these (ACC and/or DLPFC) brain regions.

In contrast to acute fatigue, which was related to increased EEG alpha and theta power densities [34], [35], moderate- to long-term fatigue was associated with decreased alpha (and in part theta) power densities. Since decreased alpha power activity has been related to sleepiness [13], this type of fatigue might have similar physiological characteristics to sleepiness in the central nervous system rather than acute fatigue. However, this similarity appears to be limited. The alpha attenuation coefficient, which is an index of sleepiness [13], was not associated with moderate- to long-term fatigue and theta power density was positively related to sleepiness [13], [36] although our results showed a negative association. EEG differences between sleepiness and moderate- to long-term fatigue were apparent, and thus the decreased alpha (and theta) EEG power densities might be specific to this type of fatigue.

Whereas local synchronization in the brain during information processing has been shown to evolve in the gamma frequency range, synchronization between neighboring cortices during multi-modal information processing evolves in the beta frequency range, and long range interactions during high-level information processing such as visuospatial attention and working memory evolves in the alpha and theta frequency ranges [37]. Since high-level information processing is associated with the alpha (and theta) power densities, decreased alpha (and theta) power densities under the condition of moderate- to long-term fatigue indicate deteriorations of the high-level information processing in the central nervous system. Our results from the cognitive tasks support this assumption.

In conclusion, we identified moderate- to long-term fatigue-related changes in cognitive performance and EEG variables: The percent error of Task 2 for Stroop trials was positively associated with the copy number of saliva HHV-6 DNA, and EEG power densities (especially the alpha power density) during the eye-closed condition were negatively associated with the saliva HHV-6 DNA level, though not during the eye-opened condition. Our findings provide new perspectives on the neural mechanisms underlying moderate- to long-term fatigue as well as evaluation methods for this type of fatigue. The present study reports data from a limited number of participants and to generalize these results, studies involving a larger number of participants will be needed.
